# Establishment and Initial Experience of Clinical FLASH Radiotherapy in Canine Cancer Patients

**DOI:** 10.3389/fonc.2021.658004

**Published:** 2021-05-13

**Authors:** Elise Konradsson, Maja L. Arendt, Kristine Bastholm Jensen, Betina Børresen, Anders E. Hansen, Sven Bäck, Annemarie T. Kristensen, Per Munck af Rosenschöld, Crister Ceberg, Kristoffer Petersson

**Affiliations:** ^1^ Medical Radiation Physics, Department of Clinical Sciences, Lund University, Lund, Sweden; ^2^ Department of Veterinary Clinical Sciences, University of Copenhagen, Frederiksberg, Denmark; ^3^ Veterinärhuset Öresund, Limhamn, Sweden; ^4^ Department of Biotherapeutic Engineering and Drug Targeting, DTU Health Tech, Kgs, Technical University of Denmark, Lyngby, Denmark; ^5^ Radiation Physics, Department of Hematology, Oncology and Radiation Physics, Skåne University Hospital, Lund, Sweden; ^6^ Department of Oncology, Oxford Institute for Radiation Oncology, University of Oxford, Oxford, United Kingdom

**Keywords:** flash, ultra-high dose rate, radiotherapy, radiation oncology, canine cancer patients, normal tissue, dosimetry

## Abstract

FLASH radiotherapy has emerged as a treatment technique with great potential to increase the differential effect between normal tissue toxicity and tumor response compared to conventional radiotherapy. To evaluate the feasibility of FLASH radiotherapy in a relevant clinical setting, we have commenced a feasibility and safety study of FLASH radiotherapy in canine cancer patients with spontaneous superficial solid tumors or microscopic residual disease, using the electron beam of our modified clinical linear accelerator. The setup for FLASH radiotherapy was established using a short electron applicator with a nominal source-to-surface distance of 70 cm and custom-made Cerrobend blocks for collimation. The beam was characterized by measuring dose profiles and depth dose curves for various field sizes. Ten canine cancer patients were included in this initial study; seven patients with nine solid superficial tumors and three patients with microscopic disease. The administered dose ranged from 15 to 35 Gy. To ensure correct delivery of the prescribed dose, film measurements were performed prior to and during treatment, and a Farmer-type ion-chamber was used for monitoring. Treatments were found to be feasible, with partial response, complete response or stable disease recorded in 11/13 irradiated tumors. Adverse events observed at follow-up ranging from 3-6 months were mild and consisted of local alopecia, leukotricia, dry desquamation, mild erythema or swelling. One patient receiving a 35 Gy dose to the nasal planum, had a grade 3 skin adverse event. Dosimetric procedures, safety and an efficient clincal workflow for FLASH radiotherapy was established. The experience from this initial study will be used as a basis for a veterinary phase I/II clinical trial with more specific patient inclusion selection, and subsequently for human trials.

## Introduction

FLASH radiotherapy (FLASH-RT) has emerged as a treatment modality with the potential to revolutionize the field of radiotherapy. The radiation dose is delivered in a fraction of a second, which is considerably faster than conventional radiotherapy, where the dose rate is typically a few Gy per minute. In 2014, Favaudon et al. presented the concept of FLASH (1), showing that delivering the dose at ultra-high dose rates resulted in reduced normal tissue toxicity in mice compared to delivering the dose at conventional dose rates, while being equally effective in killing cancer cells. Since then, several *in vivo* studies have been conducted confirming the sparing effect (2–7) and the retained tumor control (6–8). One veterinary trial has been published on FLASH-RT, including six feline cancer patients with squamous cell carcinoma of the nasal planum, also proving the potential of this technique (5). So far, one human treatment has been reported, with promising results (9). This patient suffered from a CD30+ T-cell cutaneous lymphoma and the treatment was administered as a single fraction of 15 Gy.

These intriguing findings have resulted in an increased interest in advancing FLASH-RT towards clinical trials (7, 10, 11). The progression towards this goal has been limited by the low availability of accelerators that can deliver ultra-high dose rate electrons in a clinical setting. Most of the studies mentioned above, including the first human treatment, have been conducted in research environments intended for preclinical experiments, with accelerators that are not designed for medical use (1–5, 7–9, 12). However, it has recently been shown, by our group and others, that clinical linear accelerators can be modified to deliver the dose rates needed to observe a FLASH effect (13–15). Our group has modified an Elekta Precise linear accelerator (14) so that it can operate at dose rates of 400-500 Gy/s at a source-to-surface distance of 70 cm. The possibility to perform FLASH studies using clinical linear accelerators opens up for more widespread research in this area, and facilitates translation into clinical studies.

To further explore the potential of FLASH-RT, a feasibility and safety study of FLASH-RT in canine cancer patients with spontaneous superficial solid tumors or microscopic residual disease using the electron beam of our modified clinical linear accelerator was initiated. Radiotherapy in canine cancer patients is well documented as a standard of care treatment modality for multiple tumor pathologies (16). However, in Europe, radiotherapy is used less commonly for treatment of veterinary cancer patients compared to the human situation, mainly due to lack of availability, cost and the need for multiple anesthesias required for conventional fractionated radiotherapy. Companion animal cancers are comparable to their human counterparts. They develop spontaneously in an immune competent host, at similar sizes, types, biological environment, and with similar clinical approaches to diagnosis and treatment modalities used (17, 18). This allows for veterinary clinical trials with similar radiation qualities, field sizes and targets as for human patients. Therefore, companion animal cancer patients provide an opportunity for performing cross-disciplinary research that has the potential to benefit human and veterinary cancer patients alike. In a recent review article by Nolan et al., the authors describe previous translational studies, where canine cancer patients have been used to model normal tissue response, tumor oxygenation and DNA damage response, and to optimize irradiation parameters for human radiotherapy (19). Companion animal cancer patients are usually treated with radiotherapy over a period 2-4 weeks, typically 16-20 fractions with a fractional dose of 2.5-4 Gy (19), requiring multiple anesthesia sessions which may be stressful for the patient. In contrast, FLASH-RT is delivered in a single or a few fraction(s), making this modality practical and attractive for companion animal radiotherapy. Although, previous preclinical studies have shown that fractionation of FLASH treatment does not negatively affect tumor control, some have indicated that the normal tissue sparing of FLASH is lost for fractionated treatment, where the fraction dose is below 10 Gy (7, 8). For these reasons, the canine cancer patients included in the current study received single fraction FLASH-RT, a treatment modality not otherwise available to them, which also provided us important data not attainable in preclinical rodent models.

In this paper the establishment of a clinical workflow for electron FLASH-RT in canine cancer patients is presented, with the initial overall aim of describing dosimetric procedure, treatment parameters, possible adverse events and treatment responses. This is an important step in the development of a safe and efficient workflow for FLASH-RT in a clinical setting, which could inform future human clinical trials.

## Materials and Methods

### Irradiation Source

The irradiation source was a clinical Elekta Precise linear accelerator (Elekta AB, Stockholm, Sweden) with Integrity software version 1.2 temporarily modified for electron FLASH irradiation, previously described by Lempart et al. (14). The accelerator could be modified for FLASH delivery and switched back to clinical mode in a few minutes. To achieve maximal radiation output the accelerator was operated with increased electron gun filament current and without primary and secondary scattering foils. The radiation was delivered with the standard pulse structure of 3.5 µs pulses at a pulse repetition frequency of 200 Hz. To allow the accelerator to be controlled on a pulse-by-pulse basis, an in-house built electronic circuit and a microcontroller unit was used with a diode as a beam pulse detector. Due to slight day-to-day variations, the gun filament current and magneton frequency needed to be manually tuned to achieve maximum output. This was facilitated by relative measurements with an ion-chamber.

### Setup, Beam Characterization, and Dosimetric Procedure

A setup for FLASH-RT in companion animals was established using an electron applicator with a nominal source-to-applicator distance of 65 cm. For practical reasons, the source-to-surface distance was fixed at 70 cm, i.e. 5 cm distance from the distal edge of the electron applicator. Custom-made Cerrobend blocks of various sizes were created and attached to the end of the electron applicator for field collimation. To characterize the beam, dose profiles at 2 cm depth and depth dose curves (0-4.2 cm depth) were measured in a Solid Water HE phantom (Gammex Inc., Middleton, Wisconsin, USA) using dose rate independent (20) radiochromic film (GafChromic EBT-XD, Ashland Specialty Ingredients G.P., Bridgewater, New Jersey, USA). The radiochromic film batch was calibrated in a clinical 10 MeV beam, against an ion-chamber traceable to a standard laboratory for a dose range of 1-40 Gy. Dose maximum, half-value depth, therapeutic range, full width at half maximum (FWHM) and penumbra widths (80%-20%) were determined for each given field size.

**Figure 1:**
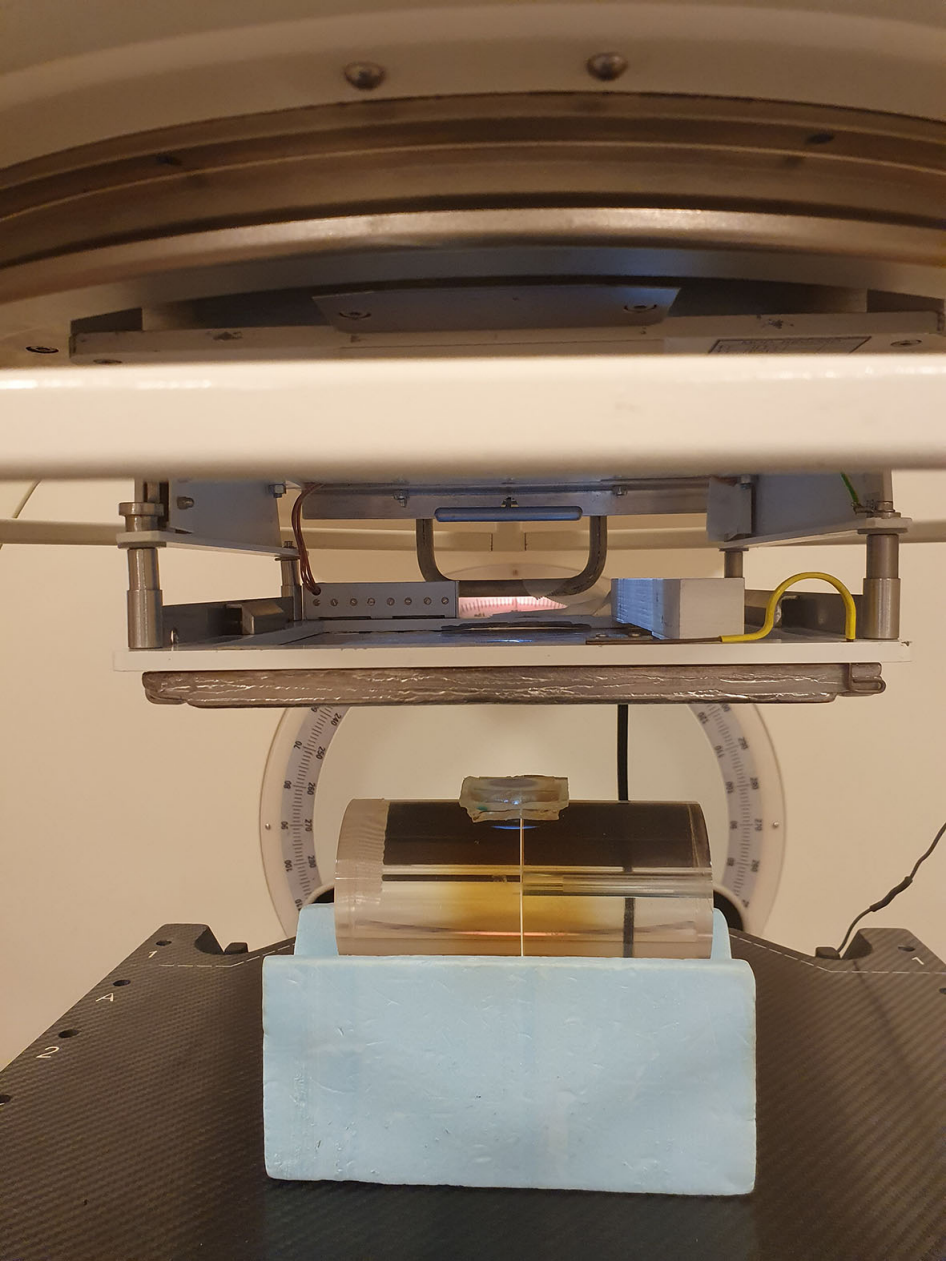
In preparation for each patient treatment, measurements with radiochromic film were performed in phantoms mimicking the treatment geometry, to determine the total dose as well as the dose-per-pulse and number of pulses to be delivered to the patient. A Farmer-type ion-chamber positioned in a customized holder in the electron applicator was used as an output monitor.

**Figure 2:**
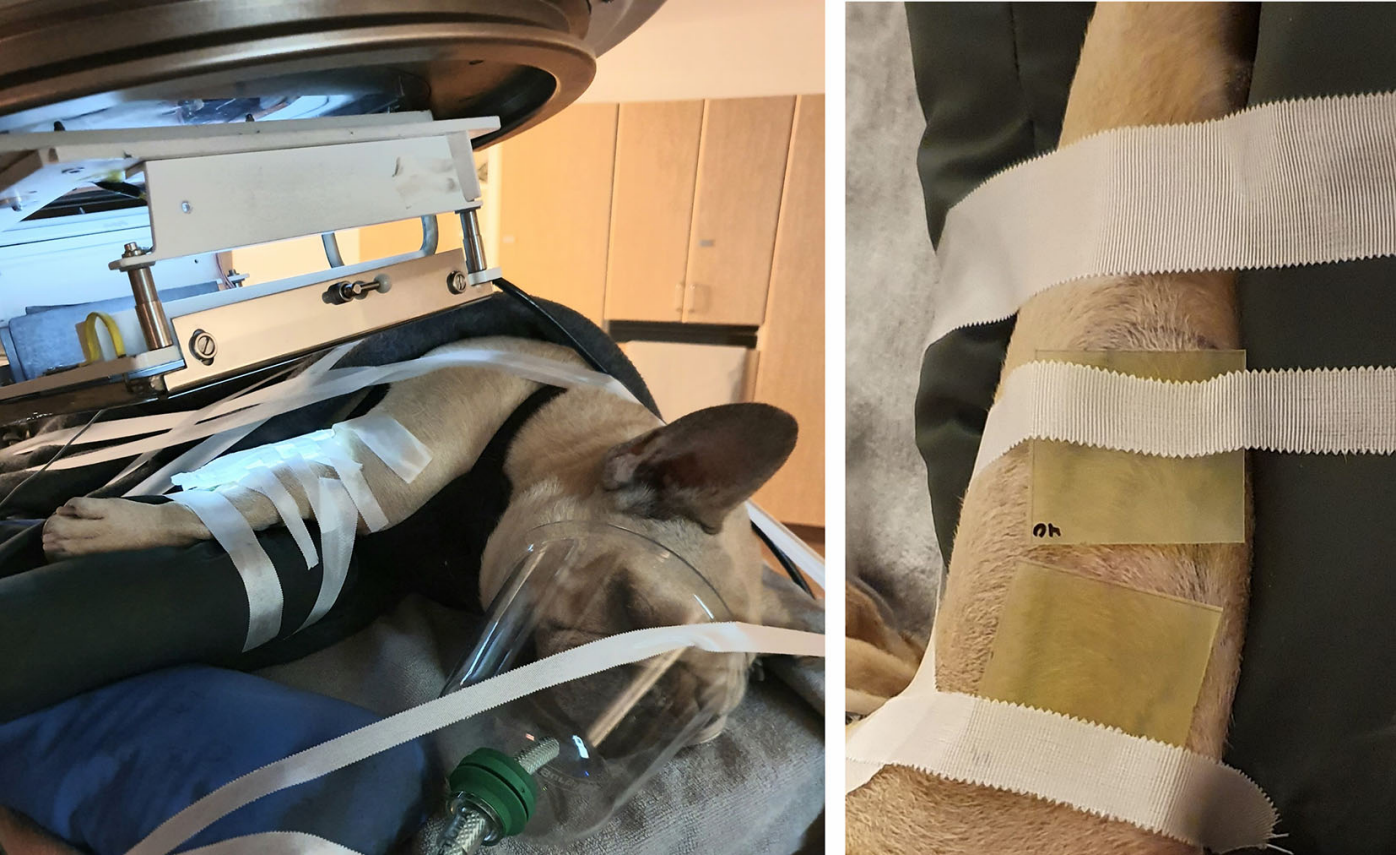
The treatment setup for patient no. 1 (left panel), with a source-to-surface distance of 70 cm and a Cerrobend plate to collimate the 8x4 cm^2^ radiation field. During the treatment, *in vivo* film measurements (right panel) were performed at the skin surface of the patient to verify the delivered dose.

Prior to each treatment, film measurements were performed in phantoms mimicking the treatment geometry (**Figure 1**) to determine the total dose as well as the dose-per-pulse (DPP) and number of pulses to be delivered to the given patient. These measurements were related to the signal from a Farmer-type ion-chamber (NE 2505/3-3A) positioned in a custom-made holder in the applicator. During treatment, the Farmer-type ion-chamber was used as an on-line monitor. In addition, EBT-XD film was used for *in vivo* dose measurements at the skin surface in the center of the beam to verify the delivered dose (**Figure 2**). The treatment volumes (≥80% of the prescribed dose) were estimated based on vertical film measurements in the solid water phantom.

### Canine Cancer Patients

The patients enrolled in this study were diagnosed during routine work up and staging with superficial solid cancers such as carcinomas, sarcomas, mast cell tumors and malignant melanomas or post-operative microscopic residual disease, where radiotherapy is the standard of care or only treatment alternative or where the owners had declined other treatment options. Diagnosis was confirmed with histopathology. Patients evaluated as poor candidates for anesthesia, such as patients with hepatic or renal insufficiency or severe heart disease, were excluded from the trial. This initial clinical feasibility and safety study included ten canine cancer patients; seven patients with a total of nine solid tumors and three patients with microscopic residual disease (**Table 1**).

**Table 1:** Description of the ten canine patients.

Patient no.	Breed	Age [years]	Weight [kg]	Target site	Diagnosis	State of disease	Post-op RT?	Est. gross tumor volume [cm^3^]
1	French Bulldog	7	13.9	Front limb	Soft tissue sarcoma	Grade 1	Yes	n/a
2	Xoloitzcuintle	10	10.3	Front limb	Soft tissue sarcoma	Grade 1	Yes	n/a
3	French Bulldog	7	12.7	Front limb	Mast cell tumor	Grade 2	Yes	n/a
4	Sibirian Husky	11	15.9	Hind limb	Plasmacytoma	n/a	No	1.3
				Hind limb	Soft tissue sarcoma	Unknown	No	11
5	Labrador Retriever	11	42.0	Eyelid	Melanoma	Amelanotic malignant	Yes^†^	0.3
				Eyelid	Melanoma	Amelanotic malignant	Yes	0.5
6	Border Collie	13	9.7	Oral (mandible)	Basosquamous carcinoma	Unknown	Yes^†^	2.6
7	Pug	8	11.6	Ear	Mast cell tumor	Unknown	No	0.5
				Eyelid	Mast cell tumor	Unknown	No	0.5
8	Cross breed	10	5.0	Oral (lip)	Mast cell tumor	Unknown	No	0.1
9	Bull Terrier	6	15.1	Abdomen	Mast cell tumor	S.C MI 5/10 HPF	No	80
10	Labrador Retriever	10	42.0	Intranasal	Squamous cell carcinoma	Unknown	No	2.0

^†^For patients no. 5 and 6, the surgery prior to FLASH-RT was performed with unclean margins and there was local recurrence prior to initiating FLASH-RT.

### Study Design

This clinical feasibility and safety study was designed as a dose-escalation trial, starting at a dose level of 15 Gy. Two-three patients were included at each dose level. A dose escalation of 5 Gy was successively performed provided no grade 3 toxicities were observed. Further consideration of different normal and tumor tissues’ sensitivity to radiotherapy was taken into account in the prescription. The primary endpoint was to evaluate the feasibility and safety of FLASH-RT with this setup, and thus this initial study was not designed to provide statistically valid results of tumor response following FLASH-RT. Though not a primary purpose of the study, a clinical benefit to the patients treated in the study was also expected.

### FLASH-RT

The ten canine cancer patients were treated during the period from March to November 2020. All tumor sites received a single beam single fraction of FLASH-RT, except for one tumor (patient no. 5) which was re-irradiated one month after the first treatment. To improve the dose distribution, treatments were planned in terms of field size and bolus thickness based on 1) clinical examination and caliper measurements, 2) CT images and/or photographs of the tumor, and 3) the beam characteristics. Tissue equivalent bolus material (Elasto-Gel EP Padding, Southwest Technologies, North Kansas City, Missouri, USA) was used for some treatments to reduce the treatment depth of the electron beam and to increase the surface dose. For oral tumors and tumors of the eyelid, an internal lead shield was used as a beam stopper to protect normal tissue. Treatment margins of 5-10 mm was used for solid tumors, and 10-20 mm for surgical scars. Set-up and treatment angle was determined when the patient was positioned on the treatment couch. Prior to treatment, patients were sedated using an adapted protocol, which enable recovery within minutes after completion of the treatment. The radiation dose was prescribed at the depth of dose maximum and was decided through discussion between medical physicists and board-certified veterinary oncologists based on tumor type and any adverse events observed in previous treatments.

### Follow-Up Procedure

Follow-up clinical evaluation occurred at approximately 7 days, 1 month and 3 months post FLASH-RT. At each follow-up evaluation, tumor response or signs of progression or relapse was evaluated together with evidence of local radiation adverse events. Tumor response was estimated based on the veterinary RECIST 1.0 criteria for patients with solid tumors (21), and disease-free interval was calculated for patients with microscopic disease. Possible adverse events were graded using the Veterinary Radiation Therapy Oncology Group (VRTOG) grading scheme for adverse events following radiotherapy (22). If the observed toxicity was found to be low-grade and well tolerated at follow-up, dose escalation to the subsequent patients was considered, taking the properties of the surrounding normal tissues into account.

### Additional Therapy

Patients already on treatment with NSAIDs for arthritic disease, continued this treatment throughout the study period. Canine cancer patients with gross mast cell tumors were treated with antihistamines and or prednisolone for approximately one week before and after radiotherapy to reduce the risk of anaphylaxis or oedema related to mast cell degranulation. Patients receiving radiotherapy to the eyelid were treated with artificial teardrops after radiotherapy to increase lubrication of the eye. One patient (patient no. 8) with unilateral submandibular lymph node metastasis diagnosed prior to radiotherapy underwent surgery to remove the affected lymph node three weeks after radiotherapy. This patients went on to receive adjuvant chemotherapy to reduce risk of further metastasis. Patient no. 5 had the irradiated eye surgically removed one month after the second dose of radiotherapy. Patient no. 9 was started on oral therapy with a tyrosine kinase inhibitor one month after radiotherapy.

### Ethics

Owners were asked to sign an informed written consent form, prior to enrollment of their animal in the study. The study was approved by the Local Ethical and Administrative Committee at Department of Veterinary Clinical Sciences, University of Copenhagen, the Danish Experimental Animals Inspectorate (2020–15–0201–00429) and the Swedish Board of Agriculture (reference number 5.2.18-02830/2020).

## Results

### Beam Characteristics

**Figure 3:**
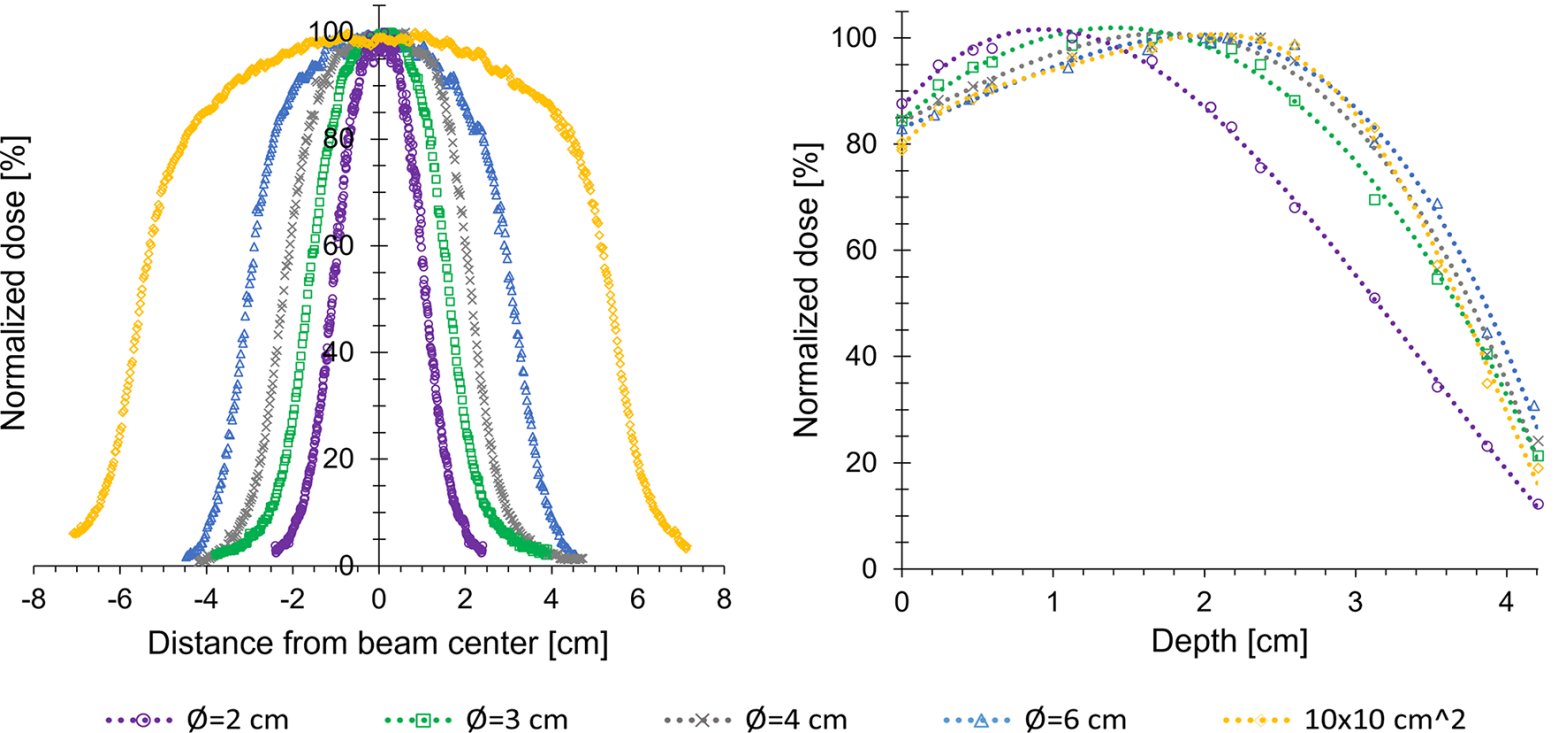
Dose profiles at 2 cm (left panel) and percentage depth dose curves (right panel) measured in a solid water phantom, for various field sizes of the Cerrobend plates fitted in the electron applicator. The therapeutic range (R_80_-value) ranged from 2.3-3.2 cm, depending on field size.

The dose profiles and the measured depth dose curves demonstrated the typical characteristics of electron beams, with a high surface dose and a rapid drop in dose beyond dose maximum (**Figure 3**). The measured dose maximums, R_50_-values, R_80_-values all increased with increasing field size, up to a field size of Ø=4 cm, after which the values were not further increased. The therapeutic range (R_80_-value) and half value depth (R_50_-value) were 2.3 cm and 3.1 cm, respectively, for the smallest field size (Ø=2 cm), and 3.1 cm and 3.8 cm for the largest field size (10x10 cm^2^). The FWHMs and penumbra widths at 2 cm depth increased with increasing field size, ranging from 2.0 to 10.3 cm and from 0.1 to 1.1 cm, respectively.

### Treatment Parameters

The prescribed dose for the ten patients ranged from 15 to 35 Gy at dose maximum, depending on tumor type, tumor size, macroscopic/microscopic disease, previously published information (5, 9), and experience from prior patient treatments. The smallest field size used for treatment was a circular field with a diameter of 2 cm, and the largest was a rectangular field of 8x4 cm^2^ (**Table 2**). Two patients (no. 4 and 7) were irradiated at two tumor sites and one patient (no. 5) was re-irradiated one month after the first treatment, which meant that a total of 13 doses were administered during the study period. Based on the Farmer-type ion-chamber signal, 92% (12/13) of the treatments were measured to be within 5% of the prescribed dose. This was subsequently confirmed by the film based *in vivo* dosimetry, showing an average agreement between prescribed and delivered skin dose of -1.8% (range -9.4% to +5.0%). Average dose rates ranged between 400-500 Gy/s and treatments were delivered in 7-16 pulses corresponding to a total treatment time ranging from 30 ms to 75 ms (**Table 2**). The instantaneous dose rates, i.e., the dose rate within each pulse, were ~7·10^5^ Gy/s.

**Table 2:** Treatment parameters for the ten canine patients.

Patient no.	Field size	Bolus [cm]	Est. volume receiving ≥80% of prescribed dose [cm^3^]	Prescribed dose [Gy]	Number of pulses	Treatment time [ms]	Average dose rate [Gy/s]
1	8 x 4 cm^2^	1.0	48	15	7	30	500
2	6 x 2 cm^2^	1.5	8.1	15	8	35	430
3	6 x 4 cm^2^	1.5	20	20	10	45	440
4	Ø = 2 cm	1.0	1.9	20	11	50	400
	Ø = 5 cm	1.0	34	25	12	55	450
5	Ø = 3 cm	1.5	4.3	25	12	55	450
	Ø = 3 cm	1.5	4.3	20^†^	9	40	500
6	Ø = 3 cm	1.5	4.3	30	15	70	430
7	Ø = 2 cm	1.0	1.9	30	15	70	430
	Ø = 2 cm	1.0	1.9	30	15	70	430
8	Ø = 2 cm	1.0	1.9	30	15	70	430
9	Ø = 5 cm	0	47	35	16	75	470
10	5 x 2 cm^2^	0	13	35	16	75	470

^†^Patient no. 5 was re-irradiated approximately 4 weeks after the first treatment.

### Follow-Up Evaluation

**Figure 4:**
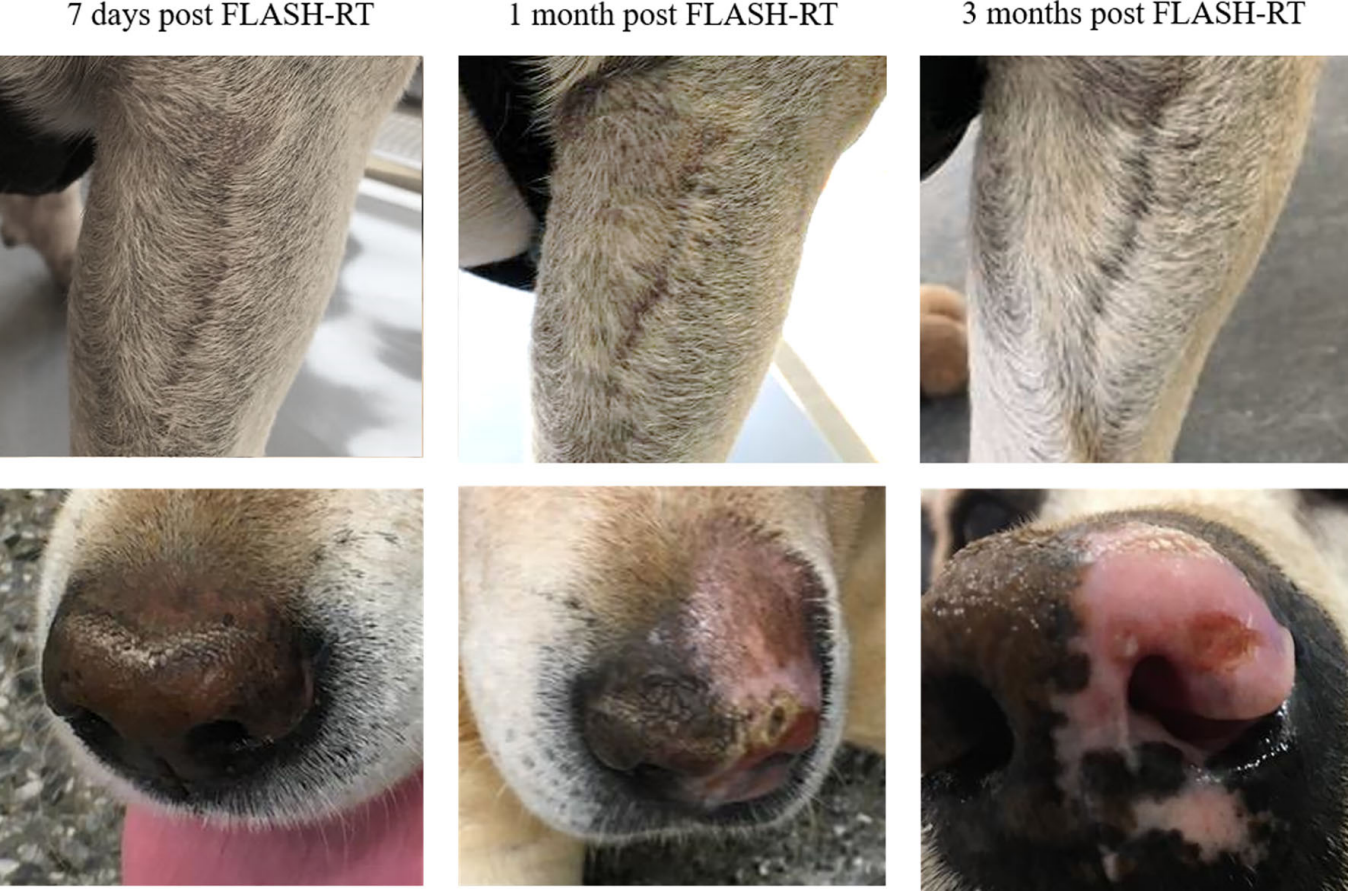
Photographs of patient no. 1 with post-operative microscopic residual disease on the front limb (top row) and patient no. 10 with intranasal squamous cell carcinoma (bottom row) at 7 days, 1 month, and 3 months post FLASH-RT.

In general, observed adverse events were mild and consisted of local alopecia, leukotricia (whiteness of the fur), dry desquamation, mild erythema or swelling (**Table 3**). However, patient no. 10, which received a 35 Gy dose to the nasal planum, had a moist desquamation affecting the part of the nasal planum included in the radiation field (**Figure 4**). This was assessed as a grade 3 skin adverse event. The desquamation started approximately 14 days after the initial therapy and had resolved completely at 1 month post radiotherapy. The irradiated skin adjacent to the nasal planum only showed evidence of mild grade 1 adverse events in terms of alopecia and mild dry desquamation. This patient received topical therapy with fucidic acid to reduce the risk of infection in the exposed dermis. Another patient (patient no. 2) developed a small ulcer in the treatment field, which was thought to be a suture reaction from previous surgery but could not be excluded as a grade 3 adverse event. This resolved with no further treatment.

**Table 3:** Adverse events (in general mild cases of local alopecia, leukotricia, dry desquamation, mild erythema or swelling) graded using the VRTOG grading scheme for adverse events following radiotherapy.

Patient no.	7 days	1 month	3 months
1	0	1	1
2	0	1^†^	0
3	0	1	1
4	0	1	1
	0	1	1
5	0	1	1
	n/a	n/a	n/a
6	0	1	1
7	0	1	1
	0	1	1
8	0	1	1
9	0	1	1
10	0	3^††^	3^††^

^†^Patient no. 2 developed a small ulcer which was thought to be a suture reaction from previous surgery.

^††^Patient no. 10 developed moist desquamation of the part of the nasal planum included in the radiation field.

The efficacy of the treatment during the follow-up period is summarized in **Table 4**. For patients with microscopic disease, no recurrence was observed during the study period. For 5/7 of the patients with solid tumors, the treatment resulted in stable disease or partial response after 1 month. Patient no. 7 showed a complete response in both tumors 3 months post irradiation. For patient no. 10 the tumor was located intranasally and response was evaluated based on clinical improvement of nasal airflow before and after therapy and visual inspection of the affected nostril. Whether the tumor response was partial or complete could not be determined clinically. Two patients (patient no. 5 and 9) had clear progressive disease after initially showing partial response to the treatment. For patient no. 7 and 8 exact measurements of tumor response were estimated partly based on clinical and visual examination rather than caliper measurement due to small size and subcutaneous or mucosal localization of tumors.

**Table 4:** Tumor response at 7 days, 1 month and 3 months post FLASH-RT, estimated based on the veterinary RECIST 1.0 criteria for patients with solid tumors, and disease-free interval for patients with microscopic disease.

Patient no.	7 days	1 month	3 months
1	NR	NR	NR
2	NR	NR	NR
3	NR	NR	NR
4	SD	SD	PR
	SD	SD	SD
5	SD^†^	PD	n/a
	SD^†^	PD	n/a
6	SD	PR	PR
7	SD	PR	CR
	SD	PR	CR
8	PR	PR	PR
9	PR	PD	PD
10	SD^††^	PR/CR^††^	CR^††^

R, Recurrence; NR, No recurrence; SD, Stable disease; PR, Partial response; CR, Complete response; PD, Progressive disease; n/a, Not applicable.

^†^The patient showed PR at 14 days followed by PD at 4 weeks after both the first and second treatment. At 4 weeks after the second treatment the tumor was surgically resected.

^††^The tumor response for patient no. 10 was based on clinical response and evaluation of nasal airflow.

## Discussion

With the setup and dosimetric procedures described in this paper, initial experience of clinical FLASH-RT in canine cancer patients using a modified clinical linear accelerator is presented. The measured depth dose curves showed that the beam can deliver >80% of the prescribed dose to a volume along the central axis ranging from the skin surface to a depth of 2-3 cm in tissue, after which the dose drops off sharply due to scattering and energy loss. These features make the beam suitable for treatments of superficial or subcutaneous tumors, but of limited use for deeper seated tumors. Hence, only superficial and subcutaneous tumors were included in this trial.

As the ten canine cancer patients included in this study were the first patients treated with FLASH-RT at our clinical accelerator, we started at a dose level that was considered as safe based on previously published information (5, 9). For one of the patients that initially showed partial response (patient no. 5), the dose administered was likely too low to control the cancer growth and hence progressive disease was seen at 4 weeks post treatment. For the second patient showing progressive disease 4 weeks post treatment (patient no. 9), the initial tumor was multilobulated and part of the tumor was seated in the abdominal musculature reaching a depth of 4.1 cm, hence it is likely that the deeper part of the tumor only received a limited part of the prescribed dose. The normal tissues showed a good tolerance during the follow-up period when irradiated with high single doses of FLASH-RT, also for the patients given 30 and 35 Gy, where mainly mild or moderate transient adverse events were observed, indicating possible further opportunities for dose escalation and extended margins to enhance the probability of tumor control. The mucous membranes in the oral cavity are generally sensitive to acute radiation side effects which can have impact on the patients’ appetite and ability to eat and have negative effect on quality of life (23). In the current study we found that FLASH-RT to the oral cavity was well tolerated and side effects were limited to grade 1 early and late side effects in terms of light injection of the mucous membranes and alteration in pigmentation. This suggests that this single fraction treatment modality can be applied to treat oropharyngeal tumors without negative impact on quality of life. This initial feasibility and safety study, with a small and heterogeneous group of participants, was not designed to provide statistically verifiable results of tumor response to FLASH-RT. A larger study is required to investigate statistical significance for the therapeutic benefit for specific cancer types and different dose levels. In addition, to investigate whether FLASH-RT is superior in sparing normal tissue compared to conventional radiotherapy, with equally effective tumor control, a comparative randomized trial with both modalities would be required in a more homogeneous patient group.

A limitation of this study is the lack of a treatment planning system for the FLASH beam to display the dose distribution in the patients. Instead, the treatment volumes were estimated based on vertical 2D film measurements in a phantom, and presented together with the estimated gross tumor volume. Using the *in vivo* film measurements, we could confirm that the prescribed dose was delivered to the patient surface with an agreement within 10%. Complex treatment geometries, such as tissue inhomogeneities, uneven air gaps and sloping surfaces, made it difficult to predict the dose distribution in advance. The ideal situation, with the electron beam impinging along the normal towards a flat surface of a homogeneous tissue, was not fulfilled in the treatments, which may have led to degrading of the dose distribution estimated to be less than 10%. Ion-chambers, which are the standard real time dosimeter in conventional radiotherapy, experience a large drop in ion collection efficiency at the ultra-high dose rates associated with FLASH radiation (24–26), making them imprecise and impractical for real time dose measurements. Therefore, the Farmer-type ion-chamber used in the current setup was functioning solely as an output monitor. Approaching human clinical trials, novel dosimetric procedures that ensure accurate delivery of the prescribed dose at FLASH irradiations, by real time dose measurements, are required. We have previously shown that the ion collection efficiency in a built-in monitor chamber can be increased by increasing the polarizing voltage over the chamber (25), and we are currently working on employing this knowledge for the setup used for our companion animal treatments by using an external monitor chamber positioned at the top of the electron applicator. We believe this approach will bring clinical dosimetry in FLASH up to the standards of conventional radiotherapy. In addition, human clinical studies will require a redundant safety system, which is an added technical challenge when using a clinical linear accelerator for FLASH-RT. When the accelerator is operated in FLASH mode, interruption of the electron beam after delivery of the desired number of pulses, is solely dependent on a diode placed in the radiation field functioning as a pulse counter. To further increase the safety during FLASH delivery, we are working on a solution where the two independent channels in the external monitor chamber can be used to interrupt the beam, similar to the method used for controlling the dose delivery in conventional radiotherapy. Furthermore, it would be favorable to be able to adjust the electron beam energy depending on the depth and size of the tumor. Currently, our FLASH beam is limited to a single energy of 10 MeV, although attempts to adjust the energy is ongoing. This would allow us to choose the treatment depth by applying an appropriate energy, and thus better exploit the advantages of an electron beam.

In addition to the companion animal cancer patients receiving superior treatment and providing valuable experience in setting up a clinical workflow for human treatments, companion animal cancer patient studies have the potential to greatly inform radiobiology studies. The mechanisms behind the FLASH sparing effect are yet to be fully understood, but the main hypothesis so far is oxygen depletion. We have previously shown *in vitro* that the FLASH effect depends on oxygen concentration (27). Due to the similarities between the tumors of companion animals and humans, also in terms of oxygen profiles (19), canine cancer patients provide an opportunity for further studying the oxygen dependence in a clinically relevant setting.

In conclusion, the first experience of electron FLASH-RT in canine cancer patients in a clinical setting is presented. Treatments were found to be feasible, with partial response, complete response or stable disease recorded in 11/13 irradiated tumors. Adverse events observed at follow-up ranging from 3-6 months were mild and consisted of local alopecia, leukotricia, dry desquamation, mild erythema or swelling. Only one patient receiving a 35 Gy dose to the nasal planum, had a grade 3 skin adverse event. Dosimetric procedures, safety and an efficient clinical workflow for FLASH-RT was established. The experience from this initial trial, in terms of a safe and efficient workflow for FLASH-RT in a clinical setting, will be used as a basis for a veterinary phase I/II clinical trial with more specific patient inclusion selection, and subsequently for human trials.

## Data Availability Statement

The original contributions presented in the study are included in the article/supplementary material. Further inquiries can be directed to the corresponding author.

## Ethics Statement

The animal study was reviewed and approved by Local Ethical and Administrative Committee at Department of Veterinary Clinical Sciences, University of Copenhagen, the Danish Experimental Animals Inspectorate (2020–15–0201–00429), and the Swedish Board of Agriculture (reference number 5.2.18-02830/2020). Written informed consent was obtained from the owners for the participation of their animals in this study.

## Author Contributions

All authors contributed to the study design. EK, MA, KB, CC, AH, and KP administered the treatments and collected the data. EK drafted the manuscript, with support from MA. All authors contributed to the article and approved the submitted version.

## Funding

This work was supported by Mrs Berta Kamprad Foundation, The Swedish Cancer Society and Skåne University Hospital’s foundations and donations.

## Conflict of Interest

The authors declare that the research was conducted in the absence of any commercial or financial relationships that could be construed as a potential conflict of interest.
